# 

**Published:** 1893-01

**Authors:** 


### INDEX TO VOLUME XLVII.

PAGE

COMMUNICATIONS.

Address, by C. L. Boyd, D.D.S. 384
Address, by J. R. Callahan, D.D.S. 1
Address, by A. A. Dillehay, D.D.S. 273
Address, by Dr. L. D. Shepard 471
Adenoid Growths and Other Diseases Incident to Primary Dentition, by Annie Felton Reynolds, D.D.S. 482
Alveolar Abscess—Plea for Extraction and Replantation in, by J. S. Marshall, M.D. 169
Alveolar Pyorrhœa — Treatment of, by Dr. D. Caracatsanis 551, 591
Anæsthesia—Local, by N. S. Hoff, D. D. S. 143
Anæsthesia—A New Apparatus for Maintaining, by Dr. Thomas Fillebrown 589
Antrum of Highmore—Treatment of Disease of the, by J. B. Van Fossen, D.D.S. 157
Arrest of Development and Decalcification of the Enamel and Dentine, by E. S. Talbot, M.D. 130
Artificial Crowns, by D. A. Allen, D.D.S. 342
Bichloride of Mercury—Experiments with, by Carrie M. Stewart, D.D.S. 417
Breath, (The), by J. Taft, D.D.S. 5
British Dental Association—Report by the Education Committee on Communications 326
Can Apical Pericementitis Occur in Connection with Roots which have been Sterilized and Filled 588

PAGE

Central Incisor—The Pedigree of the, by A. H. Thompson, D.D.S. 578
Charity, by Dr. W. S. Curtis 422
Chemistry, by Geo. B. Clement, D.D.S. 289
Chemistry in Dentistry—The Study of, by Dr. E. M. Rockwood 489
Children — Relationship of Food to Scorbutus in, by E. F. Brush, M.D. 191
Chronic Abscesses Caused by Diseased Roots — Treatment of, by Dr. Gordon White 605
Cleft Palate—Treatment of the Congenital Deformity of, by Weston A. Price, U. of M. 261
Clinical Lecture upon Hare-Lip, Cleft Palate and Epithelioma, by C. G. Darling, M.D. 73
Cocaine — Removal of the Tooth-Pulp by the Use of, by Ed. C. Briggs, M.D. 183
Cocaine—A Method of Inducing Local Anæsthesia, by Dr. Caracatsanis 492, 545
Collar Crowns and Preparation of Roots, by C. W. Jones, D.D.S. 186
Combination Fillings—A Talk on, by D. M. Clapp 375
Condition of the Dentine in Pulpless Teeth, by W. C. Barrett, M.D., D.D.S 115
Congenital Defects of the Enamel, by Dr. Otto Zsigmondy 474
Conservative Treatment of the Teeth by the Gold Process, by Dr. Peabody 279
Cranium — Pathological Conditions of the Air Cavities of the, by Dr. I. P. Wilson. 586

PAGE

Dental Caries in the Second, Third and Fourth Degrees—Treatment of, by Dr. D. Caracatsanis .................. 561
Dental Caries—Primary Cause of, by Gustavus North, A.M., D.D.S .................. 426
Dental Decay—Relation of Predisposing Causes to Active Causes of, by L. C. Ingersoll .................. 541
Dental Pathology—The General and Local in, by J. Smith Dodge, jr., M.D., D.D.S .................. 159
Dental Legislation, by H. B. Noble, D.D.S .................. 611
Dentine—Obtunding the Sensibility of, by W. C. Davis, D. D. S. .................. 500, 549
Dentine—Obtunding Sensitive, by H. Barnes, D.D.S. 16
Dentistry in Malta .................. 353
Diseases of the Gums, by John L. Gish, M.D., D.D.S ....... 81
Diseases of the Teeth and Jaws in Inebriety, by T. D. Crothers, M.D .................. 175
Disinfectants, by Carrie M. Stewart, D.D.S .................. 216
Dr. Geo. Watt—Biographical Sketch of .................. 209
Editorial Functions in Dental Journalism, by Frank W. Sage, D.D.S .................. 514
Education—Popular Dental, by L. L. Barber, D.D.S.... 350
Elements of Success, by C. W. Steele, D.D.S .................. 371
Elevator (The),—Its Principle and Utility in Extraction, by Edwin S. Cox, L.D.S .................. 377, 570
Empyema of the Maxillary Sinus, by F. L. Stillman, D.D.S .................. 53
Empyema of the Maxillary Sinus—Personal Experiences, by H. Gradle, M.D .................. 124
Epithelioma—Clinical Lecture on Hare-Lip, Cleft Palate and, by C. G. Darling, M.D .................. 73

PAGE

Ethyl Chloride as a Local Anæsthetic, by Hedwig Benson Stahlberg .................. 549
Extraction and Replantation in Alveolar Abscess—Plea for, by Dr. John S. Marshall .................. 169
Extraction of the Pulps from Teeth in a Calcified State by Trephining, by Dr. Poinsot .................. 595
Gasserian Ganglion—Removal of the, by Edmund Andrews, M.D .................. 221
Hawaiians—Among the Ancient, by Dr. J M. Whitney 574
How to Practice Dentistry Successfully, by J. L. Sweetnam, D.D.S .................. 26
Human Temperament in Relation to the Human Tooth, by Dr. Eben M. Flagg..... 584
Inebriety—Some Facts Relative to Diseases of the Teeth and Jaws in, by T. D. Crothers, M.D .................. 175
Inter-State Dental Examining Board (An), by W. E. Walker, D.D.S .................. 291
Irregularities of the Teeth—Spring Appliances for Correcting, by V. H. Jackson .................. 509, 563
Kalium Natrium—Treatment of Infected Root-Canals with, by Dr. Emil Schreier 554
Landmarks, by W. H. Whitslar, M.D., D.D.S .................. 269
Little Things, by Dr. W. H. Wright .................. 365
Mechanical Abrasion of the Teeth, by J. E. Cravens, D.D.S .................. 12
Method of Fusing Porcelain Facings to Backing and Cap, by C. G. Myers........ 512
New Remedies and Their Application, by L. P. Bethel, M.D., D.D.S .................. 346
Nomenclature Relating to Forms of the Dental Arch and Special Positions of the Teeth, by Garrett Newkirk .................. 567

PAGE
Nomenclature, Dental, by Dr. Wm. O. Kulp................ 611
Obtunding Sensitive Dentine, by H. Barnes, D.D.S........ 16
Ohio—New Dental Law of, by E. J. Waye, D.D.S.......... 338
Oral Pathology, by R. Finley Hunt .......................... 543
Orthodontia—Principles Governing the Development of Facial Contours in the Practice of, by Dr. C. S. Case.............................. 607
Oxyphosphates, by Dr. W. B. Ames.............................. 558
Possibility of Avoiding Metallic Clasps in Partial Dentures of Vulcanite, by Dr. D. Caracatsanis. ............ 564
Post-Mortem Treatment of the Dental Pulp, by Otto Arnold, D.D.S..................... 65
Primary Cause of Dental Caries, by Gustavus North, A.M., D.D.S.................... 426
Progress and Needs of Dentistry, by J. Taft, D.D.S... 105
Prosthetic Dentistry—Present and Future of, by Geo. H. Wilson, D.D.S................ 18
Pulpitis Chronica Idiopathica, by P. Macarovici, M.D.... 486
Pulpless Teeth—Condition of Dentine in, by W. C. Barrett, M.D., D.D.S............ 115
Report by the Education Committee on Communications from the British Dental Association, etc.... 326
Saliva—From a Dental Standpoint, by Carrie M. Stewart, D.D.S..................... 79
Scorbutus in Children—Relationship of Food to, by E. F. Brush, M.D..... ......... 191
Separation of the Superior Maxilla at the Symphysis, by Dr. Goddard......... 565
Soft Teeth and Galvanic Action Between Gold and Baser Metals, by Dr. Geo. W. Whitefield................ 602
Some of My Failings and the Causes, by B. F. Sims...... 428
PAGE
Staphylorraphy—Operation of, by Dr. Brophy................ 553
Status of Dentistry in the State of Mississippi under the Present Dental Laws, by C. W. Robinson, D.D.S.... 283
Status of the Art of Dentistry in Roumania, by P. Macarovici, M.D.................. 513
Sterilization of the Hands and Instruments of Dentists, by H. A. Smith, D.D.S..... 22
Surgical Engine and Its Uses, by Dr. M. H. Cryer......... 502
Teeth — Conservative Treatment by the Gold Process, by Dr. Peabody .............. 279
Teeth of the Lower Jaw at Birth, by Dr. Frank Abbott.............................. 537
The Teeth and Hair—Their Homology and Pathological Intimacy, by Dr. S. H. Guilford................... 535
Things Old, New and Useful in the Operating Room, by A. C. Hewitt, D.D.S... 313
Three Remedial Preparations, by Dr. W. E. Walker...... 285
Tin Foil for Filling Teeth, by H. L. Ambler, D.D.S....... 505
Tooth-Pulps — Methods for Obviating the Necessity of Extracting Devitalized, by Dr. W. D. Miller........ 531
Transplantation of Dental Tissues—History and Present Status of, by Dr. Louis Ottofy .......................... 597
Treatment of Infected Root-Canals with Kalium Natrium, by Dr. Emil Schreier 554
Widening Field of the Dental Profession, by Frank W. Sage, D.D.S.................. 521
What Has Dentistry to Demonstrate against the Hypothesis of Organic Evolution, by W. G. A. Bonwill, D.D.S.................... 480
What Are the Etiological Factors in the Production of the Protruded Lower Jaw? Discussion........... 609

PAGE

SOCIETY PROCEEDINGS.

Alabama Dental Association .. 384
Alumni Meeting—University of Michigan ........ ......... 614
American Dental Association.. 439
Georgia State Dental Society.. 427
Mississippi State Dental Association ........................ 273
National Association of Dental Examiners............... 444
National Association of Dental Faculties................. 441
Southern Dental Association .. 612
Vermont State Dental Society. 437
World's Columbian Dental Congress............469, 531, 573

SELECTIONS.

Abscess of the Antrum of Highmore..................... 197
Acidity of the Stomach.......... 168
Amblyopia—Due to Dental Irritation .................... 242
American Microscopical Society ............................ 200
Antiseptics.......................... ... 240
Antrum of Highmore—New Method of Penetrating..... 462
Bleaching Bones.................. 237
Bones—A Chemical Test of the Antiquity of................ 403
Burns—Application for......... 272
Carbolic Acid as an Antiseptic. 243
Carbolic Acid in Surgery........ 228
Caterpillars in Pill-boxes....... 402
Chloroform—Solidified.......... 240
Cholera—Necessity of Prevention against.................. 300
Cocaine in Surgery.............. 405
Compound Fracture of the Inferior Maxilla Treated by Wire Suture................. 149
Corrosive Sublimate in Surgery—Present Status of... 231
Culture and Professional Success ............................ 44
Dental Anæsthetics, Etc......... 235
Dentition in Infants............. 223
Do the Sick ever Sneeze?....... 463
Ethnological Characteristics of the Human Nasal Canals, by Wm. C. Braislin, M.D. 295
Expectorant........................ 238

PAGE

Explosive Powder, (An)........ 239
Eyesight—Its Care During Infancy and Youth........... 88
Frontal Headache and Iodide of Potash...................... 446
Gold—Imitation of.............. 238
Gum—Lancing.................... 241
How Can Diseases of the Nose and Throat Affect the Teeth—Abstract of a Lecture, by Edward Pynchon, M.D.................... 447
How to Rest........................ 465
Japanese Nursery Notes......... 457
Light as a Caustic................. 47
Medical Charlatans .............. 72
Medical Education—Progress in ................................ 516
Medical Hints..................... 461
Medical Temperance Association........................... 304
Microbes—Passage of Through the Skin...................... 234
Necessity for Trained and Educated Health Officials.... 458
Nervous Debility—Recipe for. 238
Overcrowded Professions....... 239
Peroxide of Hydrogen as an Aid to Diagnosis........... 198
Quack Question as Handled by the City Council of Mansfield, Ohio..................... 198
Relation of Suicide to Alcohol Consumption ................ 404
Removal of a Foreign Body (Pin), from the Throat of a Child ...................... 227
Reunion of Parts Severed from the Body...................... 235
Safe and Perilous Occupations. 41
Shock—The Nature of.......... 300
Society Work...................... 229
Some Erroneous Ideas on Ptomaines........................ 459
Suicide—Good Cause for........ 243
Sulfonal — Recent Observations on....................... 405
The Teaching Element in Congresses...................... 95
Toothache.......................... 464
Trigeminal Nerve—Treatment of Neuralgia of the.......... 302
Trional............................. 404
Two New Local Anæsthetics... 96

PAGE
Vegetable Essence as Antiseptic Dressings.................. 464
Waste Products Made Useful.. 31
Women's Medical College of Baltimore..................... 199

COLLEGE COMMENCEMENTS.

Alabama College of Dental Surgery........................ 251
American College of Dental Surgery........................ 249
Baltimore College of Dental Surgery........................ 244
Cincinnati College of Medicine and Surgery, Dental Department.................. 247
Columbian University, Dental Department............... 356
Harvard University, Dental Department.................. 358
Homeopathic Hospital College, Dental Department.. 357
Howard University, Dental Department.................. 355
Indiana Dental College......... 356
Kansas City Dental College.... 244
Louisville College of Dentistry.......................... 359
Meharry Medical College, Dental Department......... 251
Missouri Dental College........ 357
New York College of Dental Surgery........................ 248
Northwestern University, Dental School..................... 248
Ohio College of Dental Surgery............................ 249
Pennsylvania College of Dental Surgery.................... 247
Philadelphia Dental College.. 246
Royal College of Dental Surgeons of Ontario............ 250
Southern Medical College, Dental Department........ 250
University of Buffalo, Dental Department.................. 356
University of California, College of Dentistry............ 245
University of Denver, Dental Department.................. 357
University of Iowa, Dental Department.................. 358
PAGE
University of Maryland, Department of Dental Surgery............................ 245
University of Michigan, College of Dental Surgery..... 359
University of Minnesota, Dental Department............. 358
University of Pennsylvania, Department of Dentistry.. 355
Vanderbilt University, Dental Department.................. 246
Western Dental College of Kansas City................. 251

EDITORIAL.

Abscess Syringe—Dr. Dunn's Improved........................ 206
A Change........................... 259
Acknowledgement................ 312
A Correction....................... 570
Alumni Meetings................. 50
American Med. Association... 256
Biographical, Dr. J. W. Keyes. 104
Biographical, Dr. Geo. Watt... 209
Food.................................. 615
Highways, (The).................. 310
Kansas City Dental College..... 363
Meeting of the National Association of Dental Faculties............................. 49
New Dental College (A)........ 259
Ohio State Dental Examining Board.......................... 48
Ohio State Dental Society...50, 569
Pan-American Medical Congress (The),.................. 517
Registration of Foreign Diplomas in England......... 407
Sunshine and Shadow........... 413
The Specialist Before a General Medical Society........ 257
The World's Columbian Dental Congress.................. 465
The World's Fair—Accommodations......................... 205

NOTICES.

American Dental Association.. ........................... 361, 406
American Medical Association ...........................260, 306
California Dental Association. 308

PAGE
Columbian Dental Club, Chicago... 255
Illinois and Iowa State Dental Societies—Joint Meeting.. 202
International Medical Congress... 99
Kentucky State Dental Society. 203
Michigan State Dental Association—Postponement...... 305
Missouri State Dental Association... 309
Mississippi Valley Dental Society—Postponement........ 103
National Association of Dental Examiners... 406
National Association of Dental Faculties...307, 361
Ohio State Dental Society...... 568
Pan-American Medical Congress...100, 151, 202
Seventh District Dental Society of Ohio... 203
Southern Dental Association... 406
St. Louis Dental Society........ 152
The World's Columbian Dental Congress.........97, 310, 360
The World's Columbian Dental Congress—Committee on Exhibits... 203
The World's Columbian Dental Congress — Women Dentists, Etc... 252

BIBLIOGRAPHICAL.

American Medical Temperance Quarterly... 519
Compound of Dental Pathology and Dental Medicine. 311
Elements of Chemistry and Dental Materia Medica.... 101
History of the National Association of Dental Faculties... 363
Letters from a Mother to a Mother... 363
Methods of Filling Teeth....... 51
New Illustrated Dictionary of Medicine, Etc... 520
A Praetical Treatise on Mechanical Dentistry ......... 616
Prospectus of the World's Columbian Dental Congress. 153
Rise, Fall and Revival of Dental Prosthesis... 52
Series of Questions and Answers for Dental Students. 103
Transactions of the American Dental Association......... 312
Transactions of the California State Dental Society........ 104
Useful Hints for the Busy Dentist... 204

DENTAL LEGISLATION.

Dental Law of Wyoming........ 195

IN MEMORIAM.

Dr. W. W. Allport... 361
Dr. W. C. Wardlaw... 571
Dr. Geo. Watt... 207

OBITUARY.

Dr. W. W. Allport... 208
Mrs. Chas. R. Butler... 207
Dr. W. W. Dawson... 260
Dr. Arthur St. Clair Graham.. 364
Mrs. J. A. Robinson... 364
Dr. W. C. Wardlaw... 520
Dr. Geo. Watt... 156
Dr. Geo. W. McElhaney........ 572

### DIRECTORY OF DENTAL SOCIETIES.

*American Dental Association*—Meets first Tuesday in August, 1894, at Old Point Comfort. Pres., J. D. Patterson, Kansas City, Mo.; Rec. Sec., Geo. H. Cushing, Chicago.

*Southern Dental Association*—Will meet in 1894. At Old Point Comfort. Prest., B. Holly Smith, Baltimore, Md.; Secretary, D. R. Stubblefield, Nashville, Tenn.

### STATE DENTAL SOCIETIES.

*Alabama Dental Association*—Meets annually. Next meeting at Troy, on the second Tuesday of April, 1894. Pres. T. M. Allen, Birmingham; Sec. S. W. Foster, Decatur.

*California State Dental Association*—Next meeting will be held in San Francisco, July, 1894. Pres., L. A. Teague, San Francisco; Rec. Sec., W. Z. King, San Francisco; Cor. Sec., C. E. Post, San Francisco.

*Colorado State Dental Society*—Meets 1st Tuesday in June, 1894, at Glenwood Springs. Pres. P. T. Smith, Denver; Sec., Sarah May Townsend, Denver.

*Connecticut State Dental Society*—Will hold a Union Meeting in connection with the Connecticut Valley Dental Society at Hartford, third Tuesday in May, 1894. Pres. C. C. Barker, Meriden; Rec. Sec. Geo. L. Parmele, Hartford.

*Dental Society of the State of Maryland and District of Columbia*—Meets annually. Next meeting at Washington City, on the second Tuesday in October, 1893 Pres. B. F. Coy, of Baltimore: Sec. M. W. Foster, Baltimore.

*Florida State Dental Association*—Meets annually. Next meeting at Talahassee, 1st Tuesday in May, 1894. Pres, F. E. Buck, Jacksonville; Cor. Sec. E. M. Sanderson, Jacksonville

*Georgia State Dental Society*—Meets in June, 1894, at Tybee Island; Pres. N. A. Williams, Valdosta; Rec. Sec. S. H. McKee, Americus; Cor. Sec. O. H. Mc Donald, Griffin.

*Illinois State Dental Society*—The next meeting will be held at Springfield, Ill., the second Tuesday in May, 1894. Pres. Garrett Newkirk, Chicago; Sec. Louis Ottofy, Chicago.

*Indiana State Dental Society*—Will meet at Lake Maxinkuckee, on the last Tuesday of June, 1894. Pres. W. M. Hindman, Vincennes; Secretary, G. E. Hunt Indianapolis.

*Iowa State Dental Society*—Meets annually. Next meeting in Council Bluffs, on the second Tuesday in May, 1894. Pres. S. E. Hatch, Sioux City; Rec. Sec. G. W. Miller, Des Moines.

*Kentucky State Dental Association*—Meets annually. Next meeting in Richmond, Ky., June 6th, 1893. Pres., M. W. Steen, Augusta; Sec., J. H. Baldwin, Louisville.

*Kansas State Dental Association*—Pres. C. E. Esterly, Laurence; Sec., J. P. Root, Kansas City. Next meeting will be held at Topeka, last Tuesday in April, 1894.

*Maine Dental Society*—Will meet on the third Tuesday in July, 1894, in Rockland. Pres. H. A. Kelley. Portland; Sec. C. E. Bryant, Pittsfield.

*Maryland State Dental Association*—Next annual meeting will be held (place not definitely known yet,) in June, 1894. Pres., B. Holly Smith, Baltimore; Rec. Sec, W. W. Dunbracco, Baltimore; Cor. Sec., R. C. Bradshaw, Baltimore.

*Massachusetts Dental Society*—Meets annually in Boston, in June. Pres. Josiah W. Ball, Boston; Rec. Sec., E. O. Kinsman, Cambridge.

*Michigan State Dental Association*—Meets annually. Next meeting at Ann Arbor, Tuesday, June 7th, 1894. Pres., N. S. Hoff; Ann Arbor; Sec., J. Ward House, Grand Rapids. Treas. Geo. H. Mosher, Jackson.

*Mississippi State Dental Association*—Next meeting the first Tuesday in May, 1894, at Natchez, Miss. Pres. Jas. B. Askew, Vicksburg; Cor. Sec. J. D. Killian, Greenville.

*Minnesota State Society*—Pres. C. H. Stearns, Zumbrota; Sec., C. W. Jones, St. Paul. Will meet in Minneapolis, first week, in September, 1894.

*Missouri Dental Association*—Meets at Excelsior Springs, Mo., second Tuesday in July, 1894. Pres., W. E. Tucker, Springfield, Mo.; Sec., Wm. Conrad, St. Louis.

*Nebraska State Dental Society*—Meets annually. Next meeting third Tuesday in May 1893, at Beatrice; Pres. J. J. Willey, Wahoo; Sec. C. R. Tefft, Lincoln.

*New Jersey State Dental Society*—Meets annually. The next meeting will be held at Asbury Park, onthe 18th, 19th, 20th, of July, 1894. Pres. E. M. Beesley, Belvidere; Sec. C. A. Meeker, Newark.

*New Hampshire Dental Society*—Meets Annually. Next meeting will be held in Concord, N. H.; time to be decided later. Pres. W. R. Blackstone, Manchester; Sec. Burton C. Russell, Keene.

*North Carolina State Dental Society*—Meets in Durham, on the first Tuesday in May, 1894. Pres. C. A. Rominger, Reidsville; Sec., J. E. Wyche, Oxford.

*North Dakota State Dental Society*—The next meeting will be held in Grand Forks, in 1894, the exact time will be given later. Pres. B. D. McLain, of Jamestown; Sec. R. B. Foster, Grand Forks.

*Ohio State Dental Society*—Meets annually. Next meeting at Columbus, first Tuesday of December, 1893. Pres. Geo. H. Wilson, Cleveland; Sec. L. P. Bethel, Kent.

*Pennsylvania State Dental Society*—Will hold its next annual meeting at Cresson, Pennsylvania, July 11th, 1893. Pres., W. E. VanOrsdel, Sharon; Cor. Sec., C. V. Kratzer, Reading, Pa.

*Rhode Island Dental Association*—Pres, S. W. Clark, Providence; Sec., H. W. Gillett, Newport. Meets quarterly, second Tuesday in July, October, January and April. Annual Meeting second Tuesday in July, at Providence.

*South Carolina State Dental Association*—Meets annually. Pres. C. S. Patrick, Charleston; Cor. Sec. H. J. Ray, Aiken. Next meeting at Columbia, S. C., in August, 1893.

*Tennessee Dental Association*—Meets annually. Next meeting at Nashville, first Tuesday in July, 1894. Pres. W. J. Morrison, Nashville; Rec Sec. W. W. Jones, Murfreesboro; Cor. Sec. S. B. Cook, Chattanooga.

*The Dental Society of the State of New York*—Meets annually on the second Wednesday in May. Next session at Albany, 9th and 10th of May, 1894. Pres. W. W. Walker; Sec. C. S. Butler, Buffalo; Treas. John I Hart, New York.

*Vermont State Dental Society*—Meets annually. Next meeting at White River Junction, Vt., third Wednesday in March, 1894. Pres. A. J. Parker, Bellows Falls; Sec. Thos. Mound, Rutland.

*Virginia State Dental Association*—Will meet at Old Point Comfort, in 1894, Exact tine to be decided later. Pres. H. W. Campbell, Suffolk; Sec. J. Hall Moore, Richmond.

*Washington State Dental Society*—Meets Annually. Next meeting will be held at Tacoma, in the early part of May, 1894. Pres. P. H. Carlyon, Olympia; Sec., A. S. Oliver, Olympia.

*Wisconsin State Dental Society*—Meets annually. Next meeting at will be held at Janesville, Wis., the second Tuesday in July, 1893. Pres., W. C. Wendel, Milwaukee; Sec., C. A. Southwell, Milwaukee.

*West Virginia State Society.*—The annual meeting will be held at Parkersburg, on the first Wednesday of October, 1893. Pres. J. N. Mahan, Charleston; Sec. Geo. I. Keener, Morgantown.

### LOCAL SOCIETIES.

*American Academy of Dental Science*—Meets annually in Boston, Mass., on the first Wednesday in October. Pres. J. H. Batchelder; Rec. Sec. E. E. Hopkins; Cor. Sec. E. B. Hitchcock, Boston.

*Buffalo Dental Association*—Meets last Monday evening of each month. Annual meeting in June. Pres. F. H. Boswell; Sec. J. A. Stackhouse.

*Brooklyn Dental Society and Second District Society United*—Meets quarterly—Pres. T. H. Holly; Rec. Sec. A. N. Russell, Brooklyn.

*Central Dental Association of Northern New Jersey*—Meets bi-monthly; and annually. The next annual meeting will be held about the middle of February, 1894. Pres. Harvey Iredell, New Brunswick; Sec. Wm. L. Fish, Newark.

*Chicago Dental Society*—Meets monthly. Pres. J. W. Wassall; Sec. L. L. Davis, Cor. Sec. Geo. J. Dennis.

*Chicago Dental Club*—Meets on the fourth Monday of each month. Pres. A. B. Freeman; Sec. C. Stoddard Smith.

*Cleveland Dental Society.*—Meets the first Monday of each month. Pres. S. B. Dewey; Sec. Martha J. Robinson.

*Connecticut Valley Dental Society*—Meets annually. Next meeting at Hartford, May, 1894. Pres. H. Rider, Danbury, Mass.; Sec. Geo. A. Maxfield, Holyoke, Mass.

*Detroit Dental Society*—Meets monthly, Pres. E. C. Moore, Sec. H. C. Raymond.

*Eighth District Dental Society, N. Y.*—Next annual meeting April 24th, 1894, in Buffalo. Pres. F. J. Burkhart, Batavia; Sec., W. V. Grove, Buffalo.

*East Tennessee Dental Association*—Meets annually. Next meeting will be held at Athens, Tenn., in August, 1893. Pres. U. D. Billmeyer, Chattanooga; Sec. and Treas. A. W. Palmer, Chattanooga.

*Fifth District Dental Society of N. Y.*—Meets semi-annually. Next meeting in Syracuse, October, 24-26. Pres. R. F. Jones, Utica; Sec. T. B. Mason, Phoenix.

*First District Dental Society of the State of New York*—Meets annually. The next meeting will be held on the second Tuesday of October, 1892. Pres., Wm. Carr, New York City; Sec., Benj. C. Nash, New York City.

*First District Dental Society of Illinois*—Next meeting will be held in Peoria, second Tuesday in September, 1893. Pres. O. M. Daymunde, of Monmouth; Sec. W. O. Butter, La Harpe.

*Knoxville City Dental Society*—Meets monthly. President, J. T. Cazier; Sec. A. J. Cottrell.

*Mississippi Valley Dental Society*—Meets annually at Cincinnati, next Meeting March, 1894. Pres., O. N. Heise, Cincinnati; Sec. H. T. Smith, Cincinnati; Cor. Sec. H. C. Matlack Cincinnati, O.

*New England Dental Society*—Meets annually. Next meeting first Thursday in Oct., 1892, in Boston Mass. Pres. J. F. Adams, Worcester, N. H.; Sec. E. O. Kinsman, Cambridge, Mass.

*Northern Illinois Dental Society*.—Meets annually. Next meeting at Aurora, in October, 1894. Pres. E. R. Warner, Chicago; Sec. J. W. Cormany, Mt. Carroll.

*Northern Ohio Dental Association*—Meets annually. Next meeting at Put-in-Bay, O., on the 2d Tuesday in May, 1894. Pres. S. B. Dewey, Cleveland; Rec. Sec. F. H. Knowlton, Akron; Cor. Sec. J. F. Dougherty, Canton.

*Northwestern Dental Association* (embraces portions of Dakota, Minnesota and Manitoba) meets annually. Next meeting at Detroit, Dak. on the fourth Tuesday in July, 1892. Pres. J. W. Cloes, Jamestown, Dak. Sec. S. J. Hill, Fargo, Dak.

*Odontological Society of Pennsylvania*—Meets on the first Saturday evening of each month, at 8 o'clock p. m., at 13th and Arch sts; Pres. Jas. Truman, Rec. Sec. A W. Deane.

*Odontological Society of New York City*—Meets the third Tuesday of each month. Pres. Wm. Jarvie; Rec. Sec. E. T. Payne.

*Odontological Society of Cincinnati*—Meets monthly. Pres. G. Molyneaux; Sec. H. C. Matlack.

*Odontological Society of Chicago*—Meets monthly. Pres. G. Newkirk; Sec. E. Noyes.

*Pennsylvania Association of Dental Surgeons*—Meets second Tuesday of each month. Pres. H. E. Roberts; Cor. Sec. Theo. F. Chupein.

*Pittsburg Dental Society*—Meets the last Tuesday Evening of each month. Pres. J. B. Williams, Homestead; Sec. N. L. Reinecke.

*San Francisco Dental Society*—Meets the second Monday evening of each month. Pres. W. A. Knowles; Sec. Chas. Morffew; Treas. J. J. Birge.

*Sixth District Dental Society of N. Y.*—Meets semi-annually. The annual meeting will be held the first week in May, 1893. Pres., Chas. W. McCall, Binghamton; Sec., T. B. Fuller, Binghamton.

*Seventh District Dental Society of Ohio*—Meets annually. Next meeting in May, 1894. Pres., O. N. Heise, Cincinnati; Sec., J. R. Callahan, Cincinnati.

*Southern Illinois Dental Society*—Meets annually. Eighth annual meeting will be held at Edwardsville, Ill., on the 3d Tuesday in October, 1893. Pres., I. G. Dickson, McLeansboro; Sec., L. B. Torrence, Chester.

*Southern Minnesota Dental Society*—Next meeting in Mankato, second Tuesday in April, 1894. Pres. M. B. Wood, Mankato; Sec. F. B. Kremer, Minneapolis.

*St. Louis Dental Society*—Meets first and third Tuesday in each month. Pres. Dr. DeCourcy Lindsley; Cor. Sec. Wm. Conrad; Rec. Sec. J. G. Pfaff.

*The Isaac Knapp Dental Coterie of Fort Wayne, Ind.*—Meets first and third Tuesday in each month. Pres., H. C. Sites; Sec., J. S. McCurdy.

*The Minneapolis Dental Society Monthly*—Pres. E. F. Clark, Sec. E. J. Morrison.

*The Tacoma Dental Society*—Meets monthly. Pres. M. C. Burns; Sec. W. E. Burkhart.

*Washington City Dental Society*—Meets 3d Tuesday of each month. Pres. J. H. P. Benson; Rec. Sec. J. Roland Walton.

were registered; seven were refused registration. Some who were not prepared for examination and desired to practice, left for other and more congenial fields than Ohio, and a few abandoned practice—at least till they should better prepare themselves for it. We can assure the Board that its labors are duly appreciated by the profession.

### Meeting of the National Association of Dental Faculties for 1893.

The next annual meeting will, in all probability, be held in Chicago in August next. This will be, in many respects, an important meeting. It will be held about the time of the World's Columbian Dental Congress, and in all probability there will be a larger attendance of the membership than ever before; and many important questions are still open for consideration by this body, and, doubtless, new ones will arise. There will also probably be quite an addition to the membership of the body. There will undoubtedly be a large number of professors and teachers from dental colleges of other countries; they should all have a cordial invitation to meet with this association, in order that its members may have the benefit of their suggestions and experience in the work of teaching and college management. The privilege of the floor should be granted to all such for the consideration of all matters of general interest. It would certainly be a great and lasting advantage if we could in this way become familiar with the methods and practices employed in the schools of other countries; they would also thus have the opportunity of becoming familiar with our methods of procedure, and utilizing them so far as they might deem it for their interest to do so.

The coming together of the respective dental teachers of the world could not otherwise than be advantageous to all, not only in its educational influence, but in the social results as well, and, doubtless, in these ways we might all in some degree be helpful to each other.

The minutes and proceedings of this Association, from its organization to the present time will be revised and codified and put in proper shape, so that its growth and progress may be readily seen and appreciated. This occasion will certainly be one of very great interest, not only to those who may be in attendance, but to the profession throughout the world.

### CONTENTS.

COMMUNICATIONS.

EDITORIAL.

Ohio State Dental Examining Board........................................ 48

Meeting of the National Association of Dental Faculties for 1893...... 49

Alumni Meetings.............................................................. 50

Ohio State Dental Society................................................... 50

Bibliographical................................................................ 51

### THE DENTAL REGISTER,

**A MONTHLY MAGAZINE DEVOTED TO THE INTERESTS OF THE**

**DENTAL PROFESSION.**

*Terms Reduced to* $2.00 *Per Year. Single Copies,* 25 *Cents.*

**EDITED BY PROF. J. TAFT, D.D.S.**

—AND—

**Published by SAM'L A. CROCKER & CO., Ohio Dental and Surgical Depot,**

The volume commences in January and closes in December.

The REGISTER being issued monthly is always fresh, therefore Indispensable to the practitioner who desires to keep posted up with the new inventions and improvements which are daily developed. Neither expense nor effort will be spared to give value and variety to its contents Articles will be illustrated with well executed engravings whenever they will add to the interest or assist to explanation.

Its extensive circulation and frequency of issue make it one of the BEST DENTAL ADVERTISING MEDIUMS in the United States.

All subscriptions must begin with January or July.

Local or special notices, five lines or less, will be inserted for $3 each insertion; over five lines, 50 cts. per line.

All advertising bills payable in advance, or at furthest, after the issue of the first number containing the advertisement. All transient advertisements to be paid for in advance.

☞CONTRIBUTIONS SOLICITED.

Exclusively Editorial Communications should be addressed to the Editor.

The latest number of the REGISTER may be ordered with or without other good at any time, and all letters should be addressed to

**SAM'L A. CROCKER & CO., Ohio Dental and Surgical Depot, Cincinnati, O.**

